# A Switch of an Idea: Simultaneous High Tibial Osteotomy and Lateral Meniscal Allograft Transplantation

**DOI:** 10.7759/cureus.2950

**Published:** 2018-07-09

**Authors:** Wonchul Choi

**Affiliations:** 1 Department of Orthopaedics, CHA University, Cha Bundang Medical Center, Sungnam, KOR

**Keywords:** lateral meniscus, meniscal allograft transplantation, high tibial osteotomy

## Abstract

High tibial osteotomy (HTO) has been regarded as an effective treatment modality for isolated medial compartment knee osteoarthritis (OA) with varus deformity. However, management for relatively young active patients with both varus-aligned medial compartment knee OA and symptomatic irreparable lateral meniscus tear can be challenging. In this situation, correction of varus alignment by HTO and restoring the function of the lateral meniscus by meniscal allograft transplantation (MAT) can be a possible solution. We present the clinical result and technical considerations for a case of varus-aligned medial compartment OA combined with lateral meniscus tear treated by simultaneously performed medial open wedge HTO and lateral MAT.

## Introduction

High tibial osteotomy (HTO) is an established procedure for the treatment of patients with medial compartmental osteoarthritis (OA) and varus alignment of the knee, particularly in young and/or active individuals. Medial open wedge HTO has become more popular due to the improvement of surgical technique and fixation devices. Conventional HTO aims to shift the mechanical axis from medial to lateral compartment of the knee in order to decrease the loading on the arthritic medial side. However, this shift may result in increased load on lateral compartment [1, 2]. Therefore, little treatment option exists for young active patients with both varus gonarthrosis and symptomatic lateral meniscus insufficiency. If a meniscal tear is not repairable, partial or total meniscectomy is often inevitable, but the meniscectomized knee is associated with early onset of knee osteoarthritis due to a decrease in the tibiofemoral contact area and an increase in joint contact pressures, especially among people who are physically active [3, 4]. Meniscal allograft transplantation (MAT) has become an alternative treatment option for relatively young and active, but symptomatic patients, instead of subtotal or total meniscectomy [5]. However, the result after combined HTO and lateral MAT has never been reported yet.

In this case report, we present a clinical result and technical considerations in simultaneous medial open wedge HTO and lateral MAT performed on a patient with combined varus-aligned medial compartment OA and irreparable lateral meniscus tear.

## Case presentation

**Figure 1:**
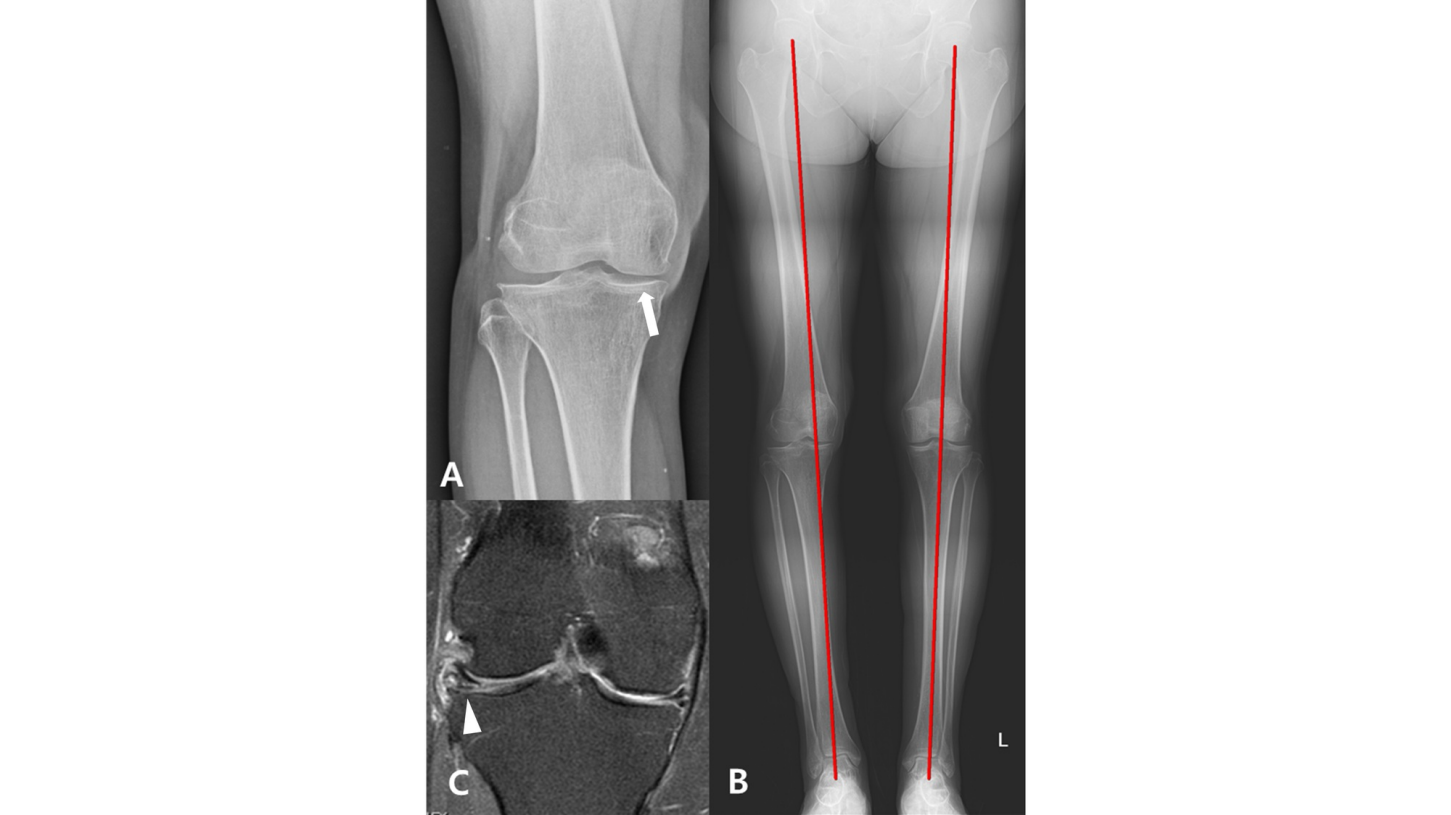
Preoperative radiographic findings. (A) Standing knee anteroposterior view showed Kellgren-Lawrence grade 2 of medial compartment osteoarthritis (arrow). (B) Weight-bearing full-length radiogram showed varus alignment in right knee and neutral alignment on left knee. (C) Magnetic resonance imaging (MRI) revealed complex tear and extrusion of lateral meniscus (arrowhead) with relatively intact lateral femoral and tibial condyles.

A 49-year-old female patient presented with complaints of worsening right knee pain since two years ago. The patient had pain when she walks, goes up the stairs, sits and gets up. The symptoms sustained even after three months of non-operative treatment. On physical examination, she had a full range of motion and had pain and tenderness on both lateral and medial joint line with positive McMurray test. No pain was observed during patellar grind and compression test. Mild to moderate degree of swelling and effusion were observed without significant instability. Plain radiographic examination showed Kellgren-Lawrence grade 2 medial compartment tibiofemoral osteoarthritis. Mechanical hip-knee-ankle axes were varus 6.5° in right knee and neutral in left knee. Posterior tibial slope angle was 4.2° and the Insall-Salvati ratio was 1.13 in right knee. Right knee magnetic resonance imaging (MRI) revealed a horizontal tear of the medial meniscus with grade 3 chondromalacia of medial femoral condyle and grade 2 chondromalacia of medial tibial condyle. Also, complex tear with extrusion of the lateral meniscus was observed with intact lateral femoral and tibial condyles. The patellofemoral joint had grade 2 chondromalacia (Figure 1).

**Figure 2:**
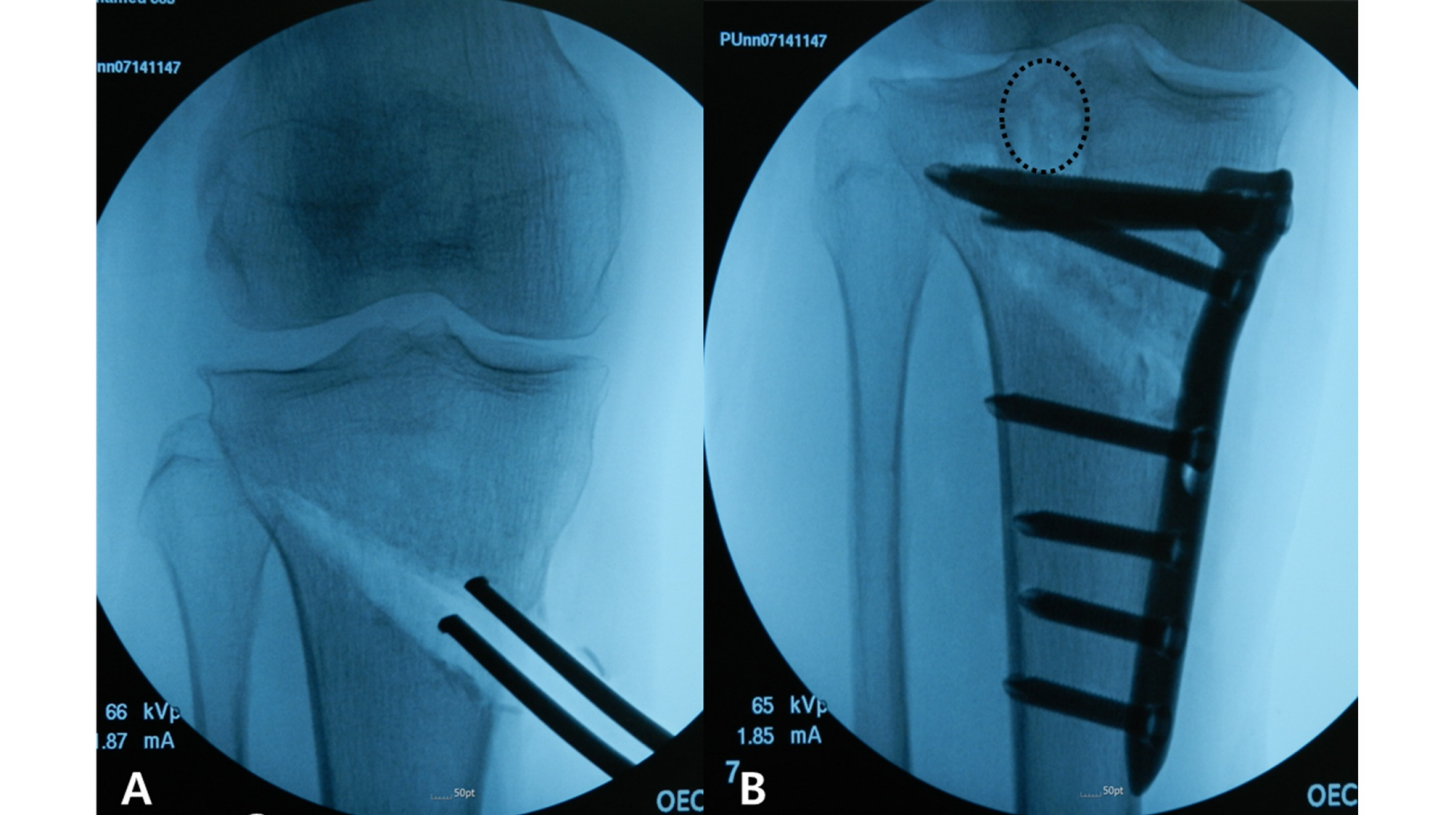
Intraoperative fluoroscopic images. (A) Medial opening wedge osteotomy was done at 1.5 cm distal to conventional osteotomy level in order to ensure enough space to fix the bony bridge of lateral meniscal allograft in proximal tibia area. (B) Anatomical locking plate fixation was done with taking care not to intrude the bony bridge of lateral meniscal allograft (dotted line) by the proximal screws.

The International Knee Documentation Committee (IKDC) score and the Western Ontario and McMaster Universities Osteoarthritis Index (WOMAC) score of the patient were 52 and 48, respectively. To address both medial compartment arthrosis and lateral meniscus tear, we planned to perform simultaneous medial open wedge HTO and lateral MAT after consulting with the patient. Knee arthroscopy was performed first, and the torn lateral meniscus was removed to within 1-2 mm of the peripheral rim, and a bleeding bed was made using a shaver. Biplanar medial open wedge HTO was performed under fluoroscopic control. Fixation of osteotomy was performed using an anatomical locking plate. After plate fixation, 5 cc of beta-tricalcium phosphate (Ca3(PO4)2) was injected into the osteotomy gap. To secure enough space for bony bridge fixation for lateral meniscal allograft in proximal tibia area, the osteotomy site and proximal screw position were made about 1.5 cm below the routine position (Figure 2).

**Figure 3:**
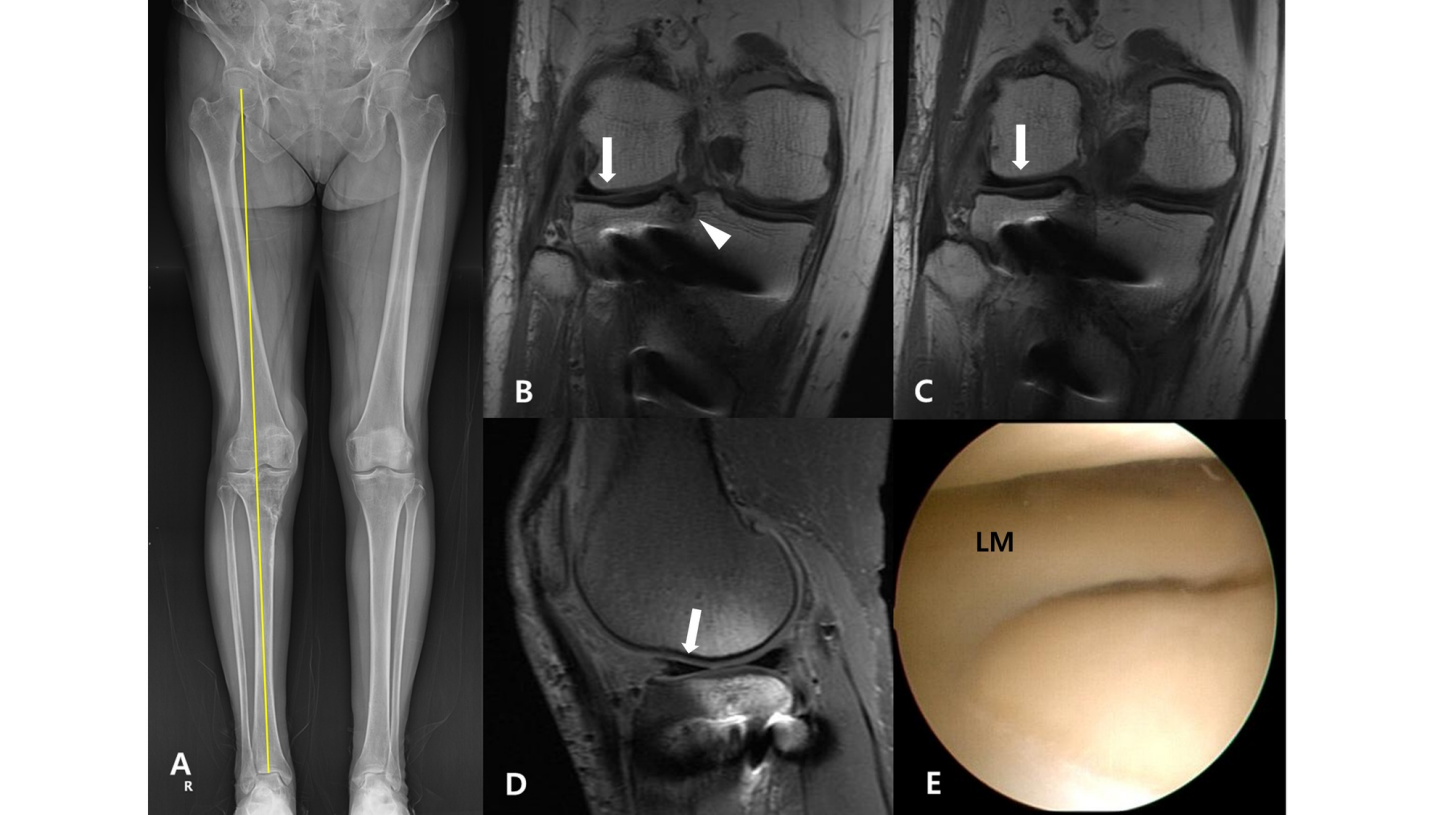
Postoperative findings. (A) Postoperative weight-bearing full-length radiogram shows union of osteotomy and correction of knee alignment to slight valgus (2.7°) angle. (B-D) Magnetic resonance imaging (MRI) taken at three months after the surgery shows intact lateral meniscus allograft (arrows) and bony bridge of the graft (arrowhead) that is unviolated by the proximal screws. (E) Second look arthroscopic examination done at postoperative one-year shows intact lateral meniscus graft.

The lateral meniscus allograft was transplanted using the keyhole technique. A keyhole slot parallel to the posterior tibial slope was made just under the lateral tibial spine. After the graft was introduced into the joint through the anterior mini-arthrotomy site, inside-out meniscal suture fixations were performed at 5 mm intervals. Bony union of osteotomy site was achieved without any complication. Radiographic measurements at postoperative one-year follow-up showed valgus 2.7° of mechanical hip-knee-ankle axis, 4.9° of posterior tibial slope, and 1.07 of Insall-Salvati Ratio. Intact lateral meniscus allograft and lateral tibiofemoral cartilage were confirmed by the follow-up knee MRI taken at postoperative three months and second-look arthroscopic examination performed at postoperative 12 months (Figure 3). Clinically, the patient had full knee range of motion and the improved IKDC and WOMAC scores of 95 and 12, respectively.

## Discussion

Uncorrected lower limb malalignment has been considered as one of the contraindications to performing MAT [6]. To address varus malalignment and medial meniscus insufficiency, combined valgus HTO and medial MAT was tried, and the favorable result has been reported [7]. However, there was no report on the result of simultaneously performed valgus HTO and lateral MAT to address the lateral meniscus insufficiency in varus knee OA.

Selection of patient is a key factor to a successful HTO. Primary or secondary medial compartment degenerative osteoarthritis is the most common indication for HTO. Lateral compartment OA is known as a relative or absolute contraindication of HTO since HTO changes the knee alignment and may increase the load on lateral compartment. The literature supports that increase in valgus angle increases the load on lateral compartment [8, 9]. Felson et al. reported that valgus alignment is a critical cause of occurrence and progression of lateral compartment OA as well as cartilage damage and lateral meniscal damage [10]. Kwon et al. evaluated the change of discoid lateral meniscus after HTO and found out that 53% of patients showed progressive degeneration of discoid lateral meniscus [11]. These findings suggest that the existing lateral meniscus problem may get worse after HTO.

In our case, medial open wedge HTO was amenable to address medial compartment OA but shifting the load by it could aggravate the combined lateral meniscus tear. Meniscectomy is often performed to address the pain related to meniscus tear; however, removing the meniscus may result in the destruction of articular cartilage and the gradual progression of knee osteoarthritis [12]. Therefore, we tried to restore the function of the lateral meniscus by MAT instead of removing it by meniscectomy.

Although the clinical result of our case was satisfactory so far, concerns remain about the survival of this procedure. An animal study in sheep has shown that standard correction with a valgus angle of 4.5° has no major detrimental effect on lateral compartment compared to 9° valgus which caused reduced cell numbers and low proliferative activities in the middle third of the vascular zone of lateral meniscus [13]. From this point of view, we tried to correct the preoperative varus alignment to only slight valgus (2.7°) angle. However, longer-term follow-up with close observation will be needed to evaluate the fate of the graft.

## Conclusions

There is a lack of treatment guidelines for the treatment of combined varus, medial compartment knee OA and symptomatic, irreparable later meniscus tear. Regarding the trustworthy outcomes and technical development of medial open wedge HTO and MAT, we suggest that simultaneous medial open wedge HTO and lateral MAT can be a viable treatment option for this troublesome situation.
